# Transitioning from paper-based to electronic health management information systems in Africa: a scoping review protocol

**DOI:** 10.1136/bmjopen-2025-114901

**Published:** 2026-05-14

**Authors:** Benson Droti, Sunny Ibeneme, Moussa Traore, Caroline Kawila, Hillary Kipruto

**Affiliations:** 1 World Health Organization Regional Office for Africa, Brazzaville, Congo; 2 Harvard T H Chan School of Public Health, Boston, Massachusetts, USA; 3 Kenya Methodist University, Nairobi, Kenya

**Keywords:** Health informatics, Information technology, Information management

## Abstract

**Abstract:**

**Introduction:**

The rising shift from paper-based to electronic health management information systems (EHMIS) among global health systems has shown promising strides over the past decade. Yet, most African health systems have continued to use paper records with attendant gaps and challenges. Most African governments have now started transitioning from paper to EHMIS but lack adequate guidance to support this shift. There is therefore a need for harmonised regional guidance to ensure that such transitions are carried out systematically and take into account country-specific contexts.

**Objective:**

The objective of this study protocol is to conduct a scoping review to generate evidence that will inform the development of a comprehensive guide to support countries in the WHO African Region in transitioning from paper-based to EHMIS.

**Methods and analysis:**

The review will follow established methodological guidance for scoping reviews as outlined by Arksey and O’Malley and further refined by Levac *et al* and the Joanna Briggs Institute, with reporting guided by the Preferred Reporting Items for Systematic Reviews and Meta-Analyses extension for Scoping Reviews checklist. A search strategy will be developed to systematically identify relevant studies from both published and grey literature sources including PubMed, Cochrane Library, Scopus and African Index Medicus. Other sources will include Google Scholar, Emerald Journal, the WHO Regional Office for Africa Library and websites of WHO, ITU and Ministries of Health. Reference lists of the retrieved published articles will be manually searched to identify additional relevant studies. Descriptive qualitative content analysis will be undertaken using the policy analysis framework and key findings will be summarised and presented using tables, charts and maps.

**Ethics and dissemination:**

This study does not involve the collection of primary data; therefore, ethical approval will not be required. On completion of the study, findings will be submitted for publication in a peer-reviewed scientific journal and will also be presented at national, regional and international conferences to support knowledge sharing and policy engagement.

**STRENGTHS AND LIMITATIONS OF THIS STUDY**

- This study follows established scoping review methodologies to systematically map evidence on transitions from paper-based to electronic health management information systems.
- The review integrates both peer-reviewed and selected grey literature sources to provide a comprehensive overview of transition approaches, challenges and enabling factors.
- Reporting the review in line with the Preferred Reporting Items for Systematic Reviews and Meta-Analyses extension for Scoping Reviews guidelines will help ensure methodological rigour
- Limiting inclusion to English-language literature may exclude relevant studies published in other languages. Future reviews could incorporate multilingual search strategies to capture a broader range of global evidence.
- The heterogeneity of electronic health management information systems definitions and reliance on available published evidence may result in some relevant experiences or unpublished implementation insights being missed.

## Background

 Recent evidence shows that African Health Information Systems (HIS) face myriad challenges with systems-wide impacts to practice and outcomes. Some of these challenges include but are not limited to the slow economic growth and poor funding for HIS, poor technological infrastructure development, data security risks, workforce capacity gaps, low professional readiness, limited stakeholder collaborations, unconducive policy environment and uncoordinated investments for HIS. In addition, many healthcare institutions in the African Region still rely on paper-based records and poorly integrated digital health records, limiting their efficiency for patient management and healthcare decision-making.^1^,^4^ The coexistence of multiple parallel information systems with paper health records has led to data fragmentation, duplication of workflows and limited use of data for planning and decision-making in most contexts.^5 6^ Thereby necessitating the need for countries to fully transition from paper records to electronic health management information systems (EHMIS).

Despite growing national interest on this topic, at the time of drafting this protocol, there were no existing syntheses that provided guidance or framework for transitioning from paper to electronic health management systems. To fill this gap, we are planning a scoping review to document the stepwise transition pathways, identify common challenges and enablers, and highlight critical knowledge gaps associated with such transitions. The consolidated evidence can inform research, policy and practice of healthcare delivery. The review will inform the development of a comprehensive guide to support countries (Algeria, Angola, Benin, Botswana, Burkina Faso, Burundi, Cameroon, Cape Verde, Central African Republic, Chad, Comoros, Ivory Coast, Democratic Republic of the Congo, Equatorial Guinea, Eritrea, Ethiopia, Gabon, Gambia, Ghana, Guinea, Guinea-Bissau, Kenya, Lesotho, Liberia, Madagascar, Malawi, Mali, Mauritania, Mauritius, Mozambique, Namibia, Niger, Nigeria, Republic of the Congo, Rwanda, São Tomé and Príncipe, Senegal, Seychelles, Sierra Leone, South Africa, South Sudan, Eswatini, Togo, Uganda, Tanzania, Zambia and Zimbabwe) in the WHO African Region to transition from paper-based to EHMIS.

Digitisation of HIS is a priority for most African countries including development partners as it facilitates the seamless flow of data, promotes appropriate use and management of resources and improves productivity among healthcare workers with system-wide impacts.^7^ According to the WHO, EHMIS are digital systems that securely store, manage, and transmit patients’ health data, including medical history, medication prescriptions, laboratory results, and billing information, to authorised healthcare providers and patients.^4 5^ Integrated EHIMS have opportunities to contribute to care coordination and systems strengthening by enhancing coverage and access to quality services. They facilitate cross-cutting programmatic support and functionalities for end-user clients, healthcare providers, data officers, and health system managers with optimal outcomes and impact.^8 9^

Studies show that innovative digital systems enable clinical decision-support systems, strengthen service delivery and improve health outcomes.^10^,^12^ Karamagi *et al* documented the impact of health facility digitisation in healthcare delivery. Their study documented that EHMIS improved health facility’s capacity to deliver patient-centred care by reducing operational costs, while expediting administrative procedures and facilitating timely and efficient care coordination.^13^,^15^ This lends credence to the study by MacLean and Titah who demonstrated how integrated EHMIS improved efficiency in patient management by facilitating lean patient management practices including real-time sharing of patient information and cost-saving consultations.^16^

Galliers *et al* documented the need for national systems to transit from traditional paper-based record keeping to integrated electronic HIS. They maintained that such transitions have opportunities to improve data accuracy, streamline processes, and facilitate data-driven decision-making across the continuum of care, as well as foster patient data privacy and information security.^617^,^19^ This lends credence to assertions from Ojo and Popoola, and Kyalo and Odhiambo-Otieno who demonstrated how such transitions facilitated a near real-time availability of health information for programme planning and monitoring, as well as enabled real-time tracking of national health indices in Nigeria and Kenya, respectively.^20 21^

Developing an EHMIS requires careful planning and adherence to basic policies and regulations, with a focus on improving health outcomes. Key considerations in establishing EHMIS according to Azzopardi-Muscat *et al*^14^ include but are not limited to—landscaping healthcare needs, ensuring regulatory compliance, promoting interoperability and integration, prioritising security and privacy, user-centred design, system testing and scalability, cost of management and effective evaluation.^9^,^11^ However, most country programmes in Africa lack the institutional capacity and operational framework to develop, adopt and institutionalise EHMIS within their systems and as such still rely on paper records for service delivery with attendant gaps and challenges.^13^,^1522^

A recent report from WHO highlighted opportunities on the use of the EHMIS and encouraged countries to have a well-functioning EHMIS in place to support the generation of reliable high-quality data for delivering quality health services that meet population health needs. This includes transitioning from paper to integrated EHMIS for service delivery.^6 23^ For this to happen, according to Heath and Porter, there is a need to strengthen existing paper records to further support the recording and data migration processes as part of the broader transition efforts to ensure comprehensive transition and institutionalisation processes. Thus, all hands must be on deck, more work on system strengthening needs to be done, and mechanisms must be put in place to support countries transitioning to EHMIS to ensure the efficient tracking of patient records, data migration and health information management. Such mechanisms will ensure that governments are accountable, responsible and committed, and will be supported to fully institutionalise EHMIS that serve the needs of the people.^24 25^

Such national enterprise transitions do not happen without challenges and impediments which vary for different contexts and countries.^26^,^29^ Although most African countries have some form of national HIS policies and strategies to inform such transitions^30^; yet there is a need for a harmonised regional guidance that highlights stepwise transition pathways, documenting potential challenges and mitigating strategies for related transitioning bottlenecks. This will include developing a regional blueprint and operational frameworks on digital health enterprise solution roll-out that takes into consideration the contextual peculiarities of the African region. Such guidance will provide a structured framework for creating effective, secure and patient-centred EHMIS that contributes to better healthcare delivery and outcomes. It will also provide clarity on the processes of setting up a digital facility-wide HIS that ensures alignment and synchronisation with health information sharing standards between and within health facilities enabled by Information and Communication Technology (ICT) best practices.

While realist reviews are useful for examining context–mechanism–outcome relationships, and understanding how specific interventions work in particular settings, the objective of this study is to map the breadth and characteristics of existing evidence on transitions from paper-based to EHMIS. Therefore, a scoping review approach was considered more appropriate, as it allows for the systematic identification and synthesis of diverse evidence, implementation experiences, and knowledge gaps across different contexts, particularly in low- and middle-income countries (LMICs). Findings from this review is expected to provide an evidence-base that can inform future in-depth research, including realist or implementation-focused reviews aimed at exploring context-specific mechanisms and outcomes of EHMIS transitions.

## Aims and objectives

### Aim

The aim of this study protocol is to conduct a scoping review to generate evidence that will inform the development of a comprehensive guide to support countries in the WHO African Region in transitioning from paper-based to EHMIS.

### Objectives

The objective of this study is to synthesise international evidence on the transition from paper-based to EHMIS and identify lessons that may inform similar digital health transformation efforts in LMIC settings with the view to generate and consolidate evidence on the following:

1. Chart the stepwise transition pathways, identify common challenges and enablers and highlight critical knowledge gaps.
2. Document system integration, data migration and quality assurance strategies as part of the broader transition processes, including strategies for troubleshooting and mitigating identified challenges.
3. Assess training and capacity building needs including documentation of case-studies and real-life examples.

## Methods

### Study design

This protocol outlines the methods and steps that will guide our scoping review on the transition from paper-based to EHMIS in Africa. A team of technical experts and researchers with experience and interest in the relevant disciplines (HIS, digital health, health system research and scoping review methodology) will design, conduct and report the scoping review. The review will be conducted following the methodological framework proposed by Arksey and O’Malley (2005), and the updated scoping review guidance provided by the Joanna Briggs Institute (JBI). A search strategy will be developed to systematically identify relevant studies. The search will include both published and grey literature sources. The following databases will be searched for published literature: PubMed, Cochrane Library, Scopus and African Index Medicus. In addition, various platforms and repositories, such as Google Scholar, Emerald Journal, the WHO Regional Office for Africa Library (AFROLIB) and websites of WHO, ITU and Ministries of Health, will be searched for published literature. Furthermore, reference lists of the retrieved published articles will be manually searched to identify additional relevant studies. Descriptive qualitative content analysis will be undertaken using the policy analysis framework of Walt and Gilson. The key findings will be summarised and presented using tables, charts and maps.^31 32^ Reporting of the review findings will be informed by the Preferred Reporting Items for Systematic Reviews and Meta-Analyses extension for Scoping Reviews (PRISMA-ScR) checklist,^33^ which will be completed and attached as an online supplemental file. We did not register this review with PROSPERO or any other registration platforms as it is not a requirement for scoping reviews to be preregistered.^34^ The full scoping is intended to start in April 2026; the projected completion date is October 2026.

### Stage 1: identifying the research questions

Considering the existing gap in literature on EHMIS transitioning frameworks, our research questions are:

1. What are the approaches, challenges and enablers involved when transitioning from paper-based to EHMIS?
2. What are the pathways for EHMIS platform set-up, data migration and database management?
3. What are the training and change management needs for institutionalising EHMIS in countries?

### Stage 2: identifying relevant documents

A comprehensive search strategy will be designed by two reviewers with supervision from the WHO/AFRO HIS technical lead, and in consultation with other research members. Key search terms that will be included are HIS, health management information systems, medical records systems, paper-based HIS, digitisation of HIS, digitalisation of HIS, transition from paper-to electronic systems, electronic medical records system, electronic health records system. Others will include paper, electronic, digital, mHealth, eHealth, health, information, management, systems, tools, interventions, framework, guidance, guideline, effectiveness, evaluation, assessment, implementation, adoption, best practices, WHO African Region, sub-Saharan Africa and a list of all WHO AFRO Member States. We will use truncation to increase yield of results.

Afterwards, the search strategy will be used for searching all eligible documents including related policy briefs, conference proceedings and grey literature. Boolean terms (‘OR’ and ‘AND’) will be used in combining search results. While ‘OR’ will be used to combine search results within the same category, ‘AND’ will be used for combining search results between different categories to help narrow down the search results.

We will focus our search on the following: PubMed, Cochrane Library, Scopus, and African Index Medicus. In addition, various platforms and repositories, such as Google Scholar, Emerald Journal, the WHO Regional Office for Africa Library (AFROLIB), websites of WHO, ITU and Ministries of Health, Digital Health Atlas and repositories for HIS. We will also search the reference list of key documents included, and will contact key subject experts listed, including persons working with MOH/ICT, WHO Country Office and WHO AFRO focal persons. A sample search strategy for Scopus is provided in online supplemental file.

### Stage 3: study selection

Identified relevant documents will be imported and deduplicated in Covidence (Covidence Systematic Review Software, 2025) and Mendeley reference management software. Two independent reviewers will further screen the source of the evidence in two stages (‘title and abstract’ and ‘full text’) using a set of predefined eligibility criteria. Discrepancies in the reviews will be discussed to reach a consensus, and unresolved disagreements will be further discussed and resolved in a wider steering group meeting involving an independent third reviewer. Articles that meet inclusion criteria will be further reviewed at the full text level. Documents selection criteria for this review will include:

#### Inclusion criteria

1. Studies that are written in English that address paper to EHMIS transition processes in the context of health facilities.
2. Systematic reviews and meta-analyses that report health facility transitions from paper to electronic health systems.
3. Documents written or published between 2005 and 2026.
4. Global scope (no restrictions).

#### Exclusion criteria

1. Materials such as opinion pieces, editorials, blogs, commentaries, non-peer reviewed articles.
2. Studies that lack methodological rigour with insufficient information.
3. Documents written or published earlier than 2005.

The terminology for describing EHMIS varies including electronic health records, electronic medical records, HIS, and health management information systems. These systems differ in scope, functionality, and levels of digital maturity. In this review, the focus is on the transition from paper-based reporting systems to EHMIS that enable systematic digital data capture, aggregation, analysis, and reporting for health system management and decision-making. Stand-alone digital health applications or isolated digital tools that do not contribute to broader HIS functionality were therefore outside the primary scope of this review.

### Stage 4: data extraction and charting

This review will report a textual and thematic synthesis of studies published between the year 2005 and the time of the search, from a global perspective. Based on study inclusion criteria, we expect most of the studies to comprise qualitative, cross-sectional and scientific reviews that must address core aspects of the reviews including transition, adoption, implementation, challenges and lessons learnt for EHMIS transitioning.

In consultation with the research team, two independent reviewers will lead the development of the data extraction form through an iterative process. The extraction tool will be piloted on at least 10% of all included articles in-line with best practices. Data extraction form refinement will continue until a consensus is reached. Any disagreements and inconsistencies will be resolved through a broader steering group meeting, with a third reviewer leading the steering group meeting. Any missing information relevant to the review process will be sought from relevant stakeholders accordingly.

As recommended by JBI guidance and Perters *et al*, this review will extract data that includes author(s), year of publication/production, country of publication/production, type of document, for example, EHMIS transition steps, EHMIS transition approaches and EHMIS transition gaps, challenges and opportunities for such transitions.^33 34^

### Stage 5: collating, analysing and presenting results

All reviewed data will be charted systematically. Full texts will be qualitatively analysed through thematic and content analysis using NVivo software and will further be summarised narratively.^35^ The PRISMA-ScR flow diagram will be used to report results as well as highlight reasons for exclusion/inclusion at the full-text stage. Details of how data were thematised and coded will be presented including potential difficulties or limitations encountered during the study in-line with Arksey and O’Malley.

### Stage 6: consultation

A consultation exercise, though not planned at this scoping review stage, may be conducted during the review if considered necessary.^36^

### Patient and public involvement

Patients or the public were not involved in the design, or conduct, or reporting, or dissemination plans of our research.

## Ethics and dissemination

This study does not involve the collection of primary data; therefore, ethical approval will not be required. On completion of the study, the results will be prepared and submitted for publication in a peer-reviewed scientific journal. Summaries of the findings will also be disseminated to relevant institutions and stakeholders. In addition, key results will be synthesised and presented at national, regional and international conferences to support knowledge sharing and policy engagement.

## Supplementary material

10.1136/bmjopen-2025-114901 online supplemental file 1

10.1136/bmjopen-2025-114901 online supplemental file 2
