# Supplementary material for: Transitioning from paper-based to electronic health management information systems in Africa: a scoping review protocol
Source: BMJ Open. 2026 May 14;16(5):e114901. doi: 10.1136/bmjopen-2025-114901 (PMC13182398; doi:10.1136/bmjopen-2025-114901)
Supplement: online supplemental file 1 [file bmjopen-16-5-s001.docx]

**Supplementary file 1: Sample search strategy for Scopus**

Database(s): Scopus 1960 to October 2026 (final search limited to 2005 – current)

Search Strategy:

| ID | Query | Documents |
| --- | --- | --- |
| #6 | (Electronic/Digital Health Information Management System OR electronic/Digital Health Information Management system Tools OR electronic/Digital Health information management system Interventions OR mHealth OR eHealth) AND (Health systems Strengthening OR Health Systems building blocks) AND (Effectiveness OR Impact OR Evaluation OR Assessment OR Implementation OR Adoption OR Barriers OR Facilitators OR Challenges OR Strategies OR Best practices) AND (Patient outcomes OR Healthcare providers OR Policy OR Resource allocation OR Health equity OR Data privacy OR Interoperability OR User acceptance OR Cost-effectiveness OR Sustainability OR Capacity building) AND (Telemedicine OR Telehealth OR Remote monitoring OR Artificial intelligence OR Machine learning OR Big data OR Wearable devices OR Mobile applications OR Electronic medical records OR Health informatics OR Health technology assessment) AND (Data security OR Data analytics OR Decision support systems OR Clinical decision support OR Health behaviour change OR Health literacy OR Digital therapeutics OR Chronic disease management OR Health communication OR Health education OR Population health management)) AND ( ( world AND health AND organization OR who AND african AND region OR africa OR sub-saharan AND africa OR "Africa South of the Sahara" ) OR (algeria OR angola OR benin OR botswana OR burkina AND faso OR burundi OR cameroon OR cape AND verde OR cabo AND verde OR central AND african AND republic OR chad OR comoros OR ivory AND coast OR cote AND d'ivoire OR democratic AND republic AND of AND the AND congo OR equatorial AND guinea OR eritrea OR ethiopia OR gabon OR gambia OR ghana OR guinea OR guinea-bissau OR kenya OR lesotho OR liberia OR madagascar OR malawi OR mali OR mauritania OR mauritius OR mozambique OR namibia OR niger OR nigeria OR republic AND of AND the AND congo OR congo OR rwanda OR são AND tomé AND príncipe OR saint AND thomas AND prince OR senegal OR seychelles OR sierra AND leone OR south AND africa OR south AND sudan OR eswatini OR togo OR uganda OR tanzania OR zambia OR Zimbabwe )) AND ( LIMIT-TO ( PUBYEAR , 2026 ) OR LIMITTO ( PUBYEAR , 2025 ) OR LIMIT-TO ( PUBYEAR , 2024 ) OR LIMIT-TO ( PUBYEAR , 2023 ) OR LIMIT-TO ( PUBYEAR , 2022 ) OR LIMIT-TO ( PUBYEAR , 2021 ) OR LIMIT-TO ( PUBYEAR , 2020 ) OR LIMIT-TO ( PUBYEAR , 2019 ) OR LIMIT-TO ( PUBYEAR , 2018 ) OR LIMIT-TO ( PUBYEAR , 2017 ) OR LIMIT-TO ( PUBYEAR , 2016 ) OR LIMIT-TO ( PUBYEAR , 2015 ) OR LIMIT-TO ( PUBYEAR , 2014 ) OR LIMIT-TO ( PUBYEAR , 2013 ) OR LIMIT-TO ( PUBYEAR , 2012 ) OR LIMIT-TO ( PUBYEAR , 2011 ) OR LIMIT-TO ( PUBYEAR , 2010 ) OR LIMIT-TO ( PUBYEAR , 2009 ) OR LIMIT-TO ( PUBYEAR , 2008 ) OR LIMIT-TO ( PUBYEAR , 2007 ) OR LIMIT-TO ( PUBYEAR , 2006 ) OR LIMIT-TO ( PUBYEAR , 2005 ) ) | 1,901 |
| #5 | ((Electronic/Digital Health Information Management System OR electronic/Digital Health Information Management system Tools OR electronic/Digital Health information management system Interventions OR mHealth OR eHealth) AND (Health systems Strengthening OR Health Systems building blocks) AND (Effectiveness OR Impact OR Evaluation OR Assessment OR Implementation OR Adoption OR Barriers OR Facilitators OR Challenges OR Strategies OR Best practices) AND (Patient outcomes OR Healthcare providers OR Policy OR Resource allocation OR Health equity OR Data privacy OR Interoperability OR User acceptance OR Cost-effectiveness OR Sustainability OR Capacity building) AND (Telemedicine OR Telehealth OR Remote monitoring OR Artificial intelligence OR Machine learning OR Big data OR Wearable devices OR Mobile applications OR Electronic medical records OR Health informatics OR Health technology assessment) AND (Data security OR Data analytics OR Decision support systems OR Clinical decision support OR Health behaviour change OR Health literacy OR Digital therapeutics OR Chronic disease management OR Health communication OR Health education OR Population health management)) AND ( ( world AND health AND organization OR who AND african AND region OR africa OR sub-saharan AND africa OR "Africa South of the Sahara" ) OR (algeria OR angola OR benin OR botswana OR burkina AND faso OR burundi OR cameroon OR cape AND verde OR cabo AND verde OR central AND african AND republic OR chad OR comoros OR ivory AND coast OR cote AND d'ivoire OR democratic AND republic AND of AND the AND congo OR equatorial AND guinea OR eritrea OR ethiopia OR gabon OR gambia OR ghana OR guinea OR guinea-bissau OR kenya OR lesotho OR liberia OR madagascar OR malawi OR mali OR mauritania OR mauritius OR mozambique OR namibia OR niger OR nigeria OR republic AND of AND the AND congo OR congo OR rwanda OR são AND tomé AND príncipe OR saint AND thomas AND prince OR senegal OR seychelles OR sierra AND leone OR south AND africa OR south AND sudan OR eswatini OR togo OR uganda OR tanzania OR zambia OR zimbabwe)) | 2,723 |
| #4 | ( world AND health AND organization OR who AND african AND region OR africa OR sub-saharan AND africa OR "Africa South of the Sahara" ) OR (algeria OR angola OR benin OR botswana OR burkina AND faso OR burundi OR cameroon OR cape AND verde OR cabo AND verde OR central AND african AND republic OR chad OR comoros OR ivory AND coast OR cote AND d'ivoire OR democratic AND republic AND of AND the AND congo OR equatorial AND guinea OR eritrea OR ethiopia OR gabon OR gambia OR ghana OR guinea OR guinea-bissau OR kenya OR lesotho OR liberia OR madagascar OR malawi OR mali OR mauritania OR mauritius OR mozambique OR namibia OR niger OR nigeria OR republic AND of AND the AND congo OR congo OR rwanda OR são AND tomé AND príncipe OR saint AND thomas AND prince OR senegal OR seychelles OR sierra AND leone OR south AND africa OR south AND sudan OR eswatini OR togo OR uganda OR tanzania OR zambia OR zimbabwe) | 202,953 |
| #3 | (Electronic/Digital Health Information System OR Digital Health Information System Tools OR Digital Health information management Interventions OR mHealth OR eHealth) AND (Health systems Strengthening OR Health Systems building blocks) AND (Effectiveness OR Impact OR Evaluation OR Assessment OR Implementation OR Adoption OR Barriers OR Facilitators OR Challenges OR Strategies OR Best practices) AND (Patient outcomes OR Healthcare providers OR Policy OR Resource allocation OR Health equity OR Data privacy OR Interoperability OR User acceptance OR Cost-effectiveness OR Sustainability OR Capacity building) | 398,623 |
| #2 | (Electronic/Digital Health Information Management System OR Digital Health information management system Tools OR Digital Health Information Management System Interventions OR Health information systems OR mHealth OR eHealth) AND (Health systems Strengthening OR Health Systems building blocks) AND (Effectiveness OR Impact OR Evaluation OR Assessment OR Implementation OR Adoption OR Barriers OR Facilitators OR Challenges OR Strategies OR Best practices)" | 534,784 |
| #1 | (Electronic OR Digital OR Computerised Health Information management Systems Tools OR Digital Health Information Management System Interventions OR mHealth OR eHealth) AND (Health systems Strengthening OR Health Systems building blocks) | 21,703,712 |
