# Supplementary material for: Transitioning from paper-based to electronic health management information systems in Africa: a scoping review protocol
Source: BMJ Open. 2026 May 14;16(5):e114901. doi: 10.1136/bmjopen-2025-114901 (PMC13182398; doi:10.1136/bmjopen-2025-114901)
Supplement: Supplementary file 3 [file bmjopen-2025-114901.reviewer_comments.pdf]

PEER REVIEW HISTORY

BMJ Open publishes all reviews undertaken for accepted manuscripts. Reviewers are asked to complete a checklist review form and are provided with free text boxes to elaborate on their assessment. These free text comments are reproduced below.

ARTICLE DETAILS

Title (Provisional)

Transitioning from Paper-Based to Electronic Health Management Information Systems in Africa: A Scoping Review Protocol

Authors

Droti, Benson; Ibeneme, Sunny; Traore, Moussa; Kawila, Caroline; kipruto, Hillary

---

VERSION 1 - REVIEW

**Reviewer**	**1**
**Name**	**Subbe, Christian**
**Affiliation**	**Bangor University, School of Medical Sciences**
**Date**	**11-Jan-2026**
**COI**	**None**

Many thanks for the opportunity to review this protocol for a scoping of literature on the transition from paper to electronic systems.

The authors emphasise a challenging situation in many African countries in relation to documentation systems. Despite the very weak evidence for the impact of electronic health records in all areas bar medication management the authors suggest that a transition is needed and that therefore the methodology of transition needs to be researched and described.

The paper describes the methodology of a scoping review but the need for the review is in my view poorly motivated and in fact a different methodology would be more appropriate.

1. It is very unclear what the boundaries of the review and its utility would be. While the review is written by a team of researchers from Africa and while the need for African countries for better healthcare records are mentioned it is not clear from the description how this would affect the review of the literature.

2. It would appear to me that the authors are really looking for results from a realist review of the literature that focuses on the challenges of implementation and inputs and outputs in low and middle income countries to understand which implementation methodology for which systems works in which situation in which environment.

3. Limiting reviews of the literature to English sources is discriminatory and in the time of Google translate outdated. Given that some of the relevant literature is expected to be in Spanish, French or indeed Chinese this is not appropriate.

4. There are a range of different terms that are used in the literature for Healthcare Information and Management Systems. It would appear to me that the authors are aiming to investigate the transition to systems with a higher level of maturity - not any system that uses digital platforms in healthcare. This requires a dedicated part in the manuscript.

There are minor problems with the written English that can be addressed at a later state.

Overall the protocol methodology seems to be superficial and I would strongly recommend to involve a methodologist.

VERSION 1 - AUTHOR RESPONSE

Reviewer: 1
Dr. Christian Subbe, Bangor University, Ysbyty Gwynedd
Comments to the Author:
Many thanks for the opportunity to review this protocol for a scoping of literature on the transition from paper to electronic systems.
The authors emphasise a challenging situation in many African countries in relation to documentation systems. Despite the very weak evidence for the impact of electronic health records in all areas bar medication management the authors suggest that a transition is needed and that therefore the methodology of transition needs to be researched and described.

Question 1. The paper describes the methodology of a scoping review but the need for the review is in my view poorly motivated and in fact a different methodology would be more appropriate.

It is very unclear what the boundaries of the review and its utility would be. While the review is written by a team of researchers from Africa and while the need for African countries for better healthcare records are mentioned it is not clear from the description how this would affect the review of the literature.

Answer 1: We want to thank the reviewer for this important comment. We agree that the scope and practical utility of the review required further clarification. The primary aim of this scoping review is to map existing evidence on the processes, challenges, and enabling factors associated with transitioning from paper-based health information systems to electronic health management information systems (EHMIS).

Although the review team is based in Africa and the study is partly motivated by the growing need to strengthen health information systems across African health systems, the review itself was designed to examine global evidence on the transition from paper-based to electronic systems. The intention is to identify implementation experiences, strategies, and lessons that

may be relevant to countries undergoing similar transitions, particularly in low- and middle-income country contexts.

To address the reviewer's concern, we have revised the manuscript to clarify:

1. The boundaries of the review, including the types of systems and transitions being examined.
2. The global scope of the literature search, while highlighting the relevance of the findings to health systems strengthening efforts in Africa and other low- and middle-income settings.
3. The practical utility of the review is to inform the development of a framework to guide countries transitioning from paper-based to electronic health information systems.

These clarifications have been incorporated into the background and objectives sections of the manuscript to better articulate the purpose and intended contribution of the review. Please see highlighted sections on page 5, 7 & 8.

Question 2. It would appear to me that the authors are really looking for results from a realist review of the literature that focuses on the challenges of implementation and inputs and outputs in low- and middle-income countries to understand which implementation methodology for which systems work in which situation in which environment.

Answer 2: We thank the reviewer for this thoughtful observation. We agree that a realist review could provide valuable insights into how and why particular implementation approaches work in specific contexts. However, the primary objective of this study is to map the breadth of available evidence on transitions from paper-based to EHMIS, and to identify key themes, approaches, and knowledge gaps across the literature.

Given this objective, a scoping review methodology was considered most appropriate, as it allows for comprehensive exploration of heterogeneous evidence and facilitates the identification of implementation experiences, challenges, and enabling factors across different settings, particularly in low- and middle-income countries.

The findings from this scoping review are intended to inform the development of a practical framework to guide the transition from paper-based systems to EHMIS, and may serve as a foundation for future studies, including more in-depth approaches such as realist reviews that examine context–mechanism–outcome relationships in greater detail.

We have clarified this rationale in the manuscript to better distinguish the purpose of the scoping review and its role in informing subsequent implementation research (see highlighted sections of page 5).

Question 3. Limiting reviews of the literature to English sources is discriminatory and in the time of Google translate outdated. Given that some of the relevant literature is expected to be in Spanish, French or indeed Chinese this is not appropriate.

Answer 3: We thank the reviewer for this important comment and agree that relevant literature on digital health and health information systems may be published in languages other than English. In this review, the inclusion of English-language publications was primarily due to practical considerations related to the research team's language capacity and the need to ensure accurate interpretation of study findings.

We acknowledge that restricting the search to English-language sources may have resulted in the exclusion of some relevant studies published in other languages. This limitation has now been explicitly acknowledged in the manuscript. Please, see highlighted sections of page 3. However, we also note that many journals indexed in major databases publish English abstracts, which allowed potentially relevant studies to be identified during the screening process.

Future research could expand the scope of the review to include non-English literature through multilingual collaboration or translation tools to capture a broader range of global experiences with transitions from paper-based to electronic health management information systems.

Question 4. There are a range of different terms that are used in the literature for Healthcare Information and Management Systems. It would appear to me that the authors are aiming to investigate the transition to systems with a higher level of maturity - not any system that uses digital platforms in healthcare. This requires a dedicated part in the manuscript.

Answer 4: We want to thank the reviewer for this valuable observation. We agree that the literature uses a wide range of terms to describe electronic health information systems, including electronic health records (EHRs), electronic medical records (EMRs), health information systems (HIS), and health management information systems (HMIS). As noted by the reviewer, these systems may also differ significantly in their levels of digital maturity and functionality.

To address this concern, we have revised the manuscript to include a dedicated section clarifying the terminology and scope of the review (see highlighted sections of page 7 & 8). In this section, we explain that the focus of the review is on the transition from paper-based systems to more mature electronic health management information systems that support structured digital data capture, management, and reporting for health system monitoring and decision-making, rather than on isolated digital tools or stand-alone digital health applications.

We have also clarified the operational definition of EHMIS used in this review and provided examples of related terms commonly used in the literature. This addition helps ensure conceptual clarity and better defines the scope of systems included in the review.

Question 5: There are minor problems with the written English that can be addressed at a later state. Overall the protocol methodology seems to be superficial and I would strongly recommend to involve a methodologist.

Answer 5: We thank the reviewer for this constructive comment. The manuscript has been carefully revised to address minor issues related to written English and to improve overall clarity and readability.

With respect to the methodological concerns, we would like to clarify that the protocol follows established methodological guidance for scoping reviews, including the frameworks proposed by Arksey and O'Malley, further refined by Levac et al., and the Joanna Briggs Institute (JBI) methodological guidance for scoping reviews. In addition, the reporting of the protocol adheres to the PRISMA extension for Scoping Reviews (PRISMA-ScR) guidelines to ensure methodological transparency and rigor.

To further strengthen the methodological description, we have revised the Methods section to provide clearer details on the search strategy, study selection process, data extraction procedures, and data synthesis approach. These revisions aim to ensure that the protocol clearly reflects the systematic and transparent processes that will be followed during the review.

We appreciate the reviewer's suggestion and have ensured that the methodological framework and procedures are now more explicitly described in the manuscript. Pls see highlighted sections of page 2.

VERSION 2 - REVIEW

**Reviewer**	**1**
**Name**	**Subbe, Christian**
**Affiliation**	**Bangor University, School of Medical Sciences**
**Date**	**18-Mar-2026**
**COI**	

I believe that this protocol does not add to the knowledge of readers. The authors aim to investigate transition from paper to digital records but for reasons only known to themselves limit the search of the literature to African countries. This means that only a very small number of studies will be returned and learning from comparable systems but other countries will be excluded.

I doubt that the publication of the present protocol adds to the literature. It is narrow, unoriginal, unusually cites only a single data-base and ignores existing knowledge about technology adoption that could have added depth to the search and value for the readers of the journal.

I am again baffled that a project that is trying to examine how to be implement new technology across a whole continent limits searches to a single language. In the year of 2026 this is discriminatory and complete and utterly unacceptable.

The revision of the paper adds little additional information to the previous version bar minor formal adjustments.

I would suggest to the authors to undertake their search, and then submit the results with the protocol as an online appendix.
